# Improving Passive Time Reversal Underwater Acoustic Communications Using Subarray Processing

**DOI:** 10.3390/s17040937

**Published:** 2017-04-24

**Authors:** Chengbing He, Lianyou Jing, Rui Xi, Qinyuan Li, Qunfei Zhang

**Affiliations:** School of Marine Science and Technology, Northwestern Polytechnical University, Xi’an 710072, China; jingly369@mail.nwpu.edu.cn (L.J.); xirui@mail.nwpu.edu.cn (R.X.); liqinyuan@mail.nwpu.edu.cn (Q.L.); zhangqf@nwpu.edu.cn (Q.Z.)

**Keywords:** underwater acoustic communications, passive time reversal, adaptive multichannel equalization, channel estimation

## Abstract

Multichannel receivers are usually employed in high-rate underwater acoustic communication to achieve spatial diversity. In the context of multichannel underwater acoustic communications, passive time reversal (TR) combined with a single-channel adaptive decision feedback equalizer (TR-DFE) is a low-complexity solution to achieve both spatial and temporal focusing. In this paper, we present a novel receiver structure to combine passive time reversal with a low-order multichannel adaptive decision feedback equalizer (TR-MC-DFE) to improve the performance of the conventional TR-DFE. First, the proposed method divides the whole received array into several subarrays. Second, we conduct passive time reversal processing in each subarray. Third, the multiple subarray outputs are equalized with a low-order multichannel DFE. We also investigated different channel estimation methods, including least squares (LS), orthogonal matching pursuit (OMP), and improved proportionate normalized least mean squares (IPNLMS). The bit error rate (BER) and output signal-to-noise ratio (SNR) performances of the receiver algorithms are evaluated using simulation and real data collected in a lake experiment. The source-receiver range is 7.4 km, and the data rate with quadrature phase shift keying (QPSK) signal is 8 kbits/s. The uncoded BER of the single input multiple output (SIMO) systems varies between $1\times 10^{-1}$ and $2\times 10^{-2}$ for the conventional TR-DFE, and between $1\times 10^{-2}$ and $1\times 10^{-3}$ for the proposed TR-MC-DFE when eight hydrophones are utilized. Compared to conventional TR-DFE, the average output SNR of the experimental data is enhanced by 3 dB.

## 1. Introduction

Because of the unique characteristics of underwater acoustic (UWA) channels, achieving reliable high-speed wireless communications over underwater acoustic channels is still a challenging task. Typical UWA channel characteristics include very limited bandwidths, time-varying multipath propagation, double-selective channel fading, and strong background noise; furthermore, large-delay spread of the multipath leads to severe inter-symbol interference (ISI) in the received signal. Efficient mitigation of multipath propagation is one of the key design goals of most UWA communication receiver algorithms. To mitigate ISI and signal fading caused by multipath propagation, spatial diversity and equalizers are widely used in high-rate underwater acoustic communications [1,2,3].

Multichannel decision feedback equalization (MC-DFE) is one efficient way to deal with ISI. MC-DFE has been successfully applied to high-rate UWA communications over the past two decades in combination with a second-order digital phase-locked loop (DPLL) [1,4]. Various adaptive algorithms are used to update the equalizer coefficients in the MC-DFE. However, for some acoustic channels with multipath spreading over several tens of symbol intervals, the MC-DFE receiver for phase-coherent communication is extremely complex, especially for multichannel receivers containing a large number of elements. At the same time, the order of the adaptive filters, which is determined by the maximum multipath delay, increases with the signaling rate, and the performance of the system is limited because of the noise enhancement and the increased sensitivity of the adaptive algorithm [2]. To better explore the advantages of the multichannel receiver, a variety of methods were investigated to improve the performance of the single-carrier communication system, including the use of a multiple-beam domain equalizer and the subarray processing approach [5,6,7,8]. Spatial precombing (beamforming) was proposed to reduce the total number of input channels (M) to a small number (*P* < *M*) for the following MC-DFE [5]. The performances of beamforming and multichannel equalization algorithms are compared comprehensively using the experimental data [6]. The results indicate that beam-domain processing can be used to improve the performance when the signal fades across the entire array. Empirical results have shown that partitioning an array with large numbers of elements into subarrays, independently processing the signals received on each subarray, and then combining the soft outputs of the processors for each subarray can lead to improved performance and reduced computational complexity [7].

Time reversal (TR) processing is a low-complexity solution for reliable high-rate underwater acoustic communications [2,9,10,11,12,13,14,15]. The well-known benefits of TR processing include temporal and spatial focusing. TR is an effective way to shorten the channel and suppress ISI, and thus reduce the receiver complexity. However, the performance of TR processing alone is saturated because of ISI caused by imperfect focusing (q(t)≠δ(t)) [2]. To eliminate with residual ISI, the passive TR is usually followed by an adaptive single-channel DFE, which is called TR-DFE [3]. TR-DFE can improve output signal-to-noise ratio (SNR) by approximately 13 dB compared to TR, as shown in [2,9]. An adaptive spatial combination with different combination coefficients on each array sensor is proposed to enhance spatial focusing and thus improve the performance of the TR-DFE [10].

In this paper, we proposes a new receiver structure which combines passive TR processing of subarrays and a low-order adaptive multichannel DFE (TR-MC-DFE) to improve the performance of conventional passive TR. The entire array with *M* elements is divided into *P* subarrays, each of which contains *K* elements (M>P), and the signals received on each subarray are independently processed by TR alone. The outputs of subarray time reversal equalizers are then processed by a low-order multichannel DFE to produce the final estimate of the transmitted data. TR-DFE can be considered a special case of TR-MC-DFE, where P=1. Consequently, TR-MC-DFE can be viewed as a generalized time reversal equalizer. It is important to recall that TR alone usually requires a minimum four-element array to achieve acceptable performance. It is also important to note that the base station underwater can support a minimum number of receivers (e.g., *M* = 8) [11]. Many works have demonstrated that exploiting the channel sparsity leads to better receiver performance [16,17,18,19,20,21]. In TR communications, accurate channel estimation is critical for performance. Different spare channel estimation algorithms, including least squares (LS), orthogonal matching pursuit (OMP) and improved proportionate normalized least mean squares (IPNLMS), have also been investigated.

The contributions of this paper include the following aspects: (1) A TR-MC-DFE method is proposed to improve the performance of conventional passive TR communication; (2) The performances of TR-DFE and TR-MC-DFE are compared and analyzed using the data collected from one lake experiment; (3) The influence of three channel estimation algorithms on the performance of the TR-MC-DFE method is analyzed and compared.

The rest of this paper is organized as follows. System model is introduced in Section 2. Section 3 first reviews the multichannel receiver algorithms, including MC-DFE and TR-DFE, and then presents the receiver structures of TR-MC-DFE. The performance of the proposed TR-MC-DFE algorithms is analyzed using the simulated data in Section 4. Section 5 illustrates the lake experiment and the communication results. Conclusions are given in Section 6.

## 2. System Model

The transmitted signal is represented in baseband form as the following equation:

(1) $$u\left(t\right)=\sum_{n}d\left(n\right)g\left(t-nT\right),$$

where d(n) are the M-ary phase shift keying (MPSK) modulated data symbols transmitted every T seconds, and g(t) is the transmitter pulse shape filter. This signal is modulated on the center frequency $f_{c}$ and transmitted over the underwater acoustic channel. The channel impulse response for a time-varying multipath underwater acoustic channel can be described by the following equation:

(2) $$h\left(\tau ,t\right)=\sum A_{p}\left(t\right)\delta \left(t-\tau_{p}\left(t\right)\right),$$

where $A_{p}\left(t\right)$ is the amplitude of path *p* and $\tau_{p}\left(t\right)$ is the path delay. On the receiver side, the Doppler is usually compensated by resampling of the incoming signal [22]. Let $y_{k}\left(t\right)$ be the received baseband signal at the *k*-th hydrophone after resampling processing as follows:

(3) $$y_{k}\left(t\right)=\sum_{n}d\left(n\right)h_{k}\left(\tau ,t-nT\right)e^{j\theta_{k}\left(t\right)}+w_{k}\left(t\right),k=1,\;...\;M,$$

where $h_{k}\left(\tau ,t\right)$ is the overall channel response of the *k*-th channel, including physical channel and transceiver filters; $w_{k}\left(t\right)$ is the additive white Gaussian noise (AWGN); and $\theta_{k}$ is the phase rotation caused by symbol timing offset and Doppler shift.

## 3. Receiver Algorithms

### 3.1. MC-DFE

**Figure 1:**
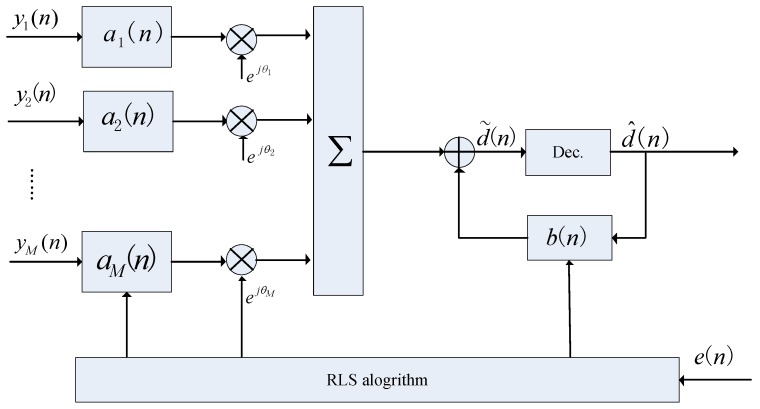
Multichannel decision feedback equalization (MC-DFE) block diagram.

The MC-DFE is very effective for removing ISI caused by multipath propagation. When the channel is unknown, the equalizer tap weights are determined by minimizing the mean square error (MSE) between the received data symbols and the recovered data symbols. Channel tracking is accomplished by combining adaptive recursive least squares (RLS) and a second-order DPLL [5]. The standard MC-DFE block diagram is displayed in Figure 1 and involves a bank of feedforward filters $a_{i}\left[n\right]$ followed by a single feedback filter b[n].

The soft output is

(4) $$\begin{array}{ccc} \tilde{d}\left(n\right) & = & p(n)-q(n)\\ & = & \sum a_{k}^{\prime }y_{k}\left(n\right)e^{-j{\hat{\theta }}_{k}\left(n\right)}-b^{\prime }\hat{d}\left(n\right). \end{array}$$

The estimated error is

(5) $$e\left(n\right)=d\left(n\right)-\tilde{d}\left(n\right),$$

where the known training sequence is d(n) when the system is in training mode, but is replaced by ${\hat{d}}_{n}$, which is the decision result of ${\tilde{d}}_{n}$ when the system is in decision feedback mode. The coefficients of feedforward and feedback filters and DPLL are updated by adaptive algorithms to minimize the output MSE = $E\{|e\left(n\right){|}^{2}\}$. The detail of MC-DFE is described in [1]. One major issue for MC-DFE is its complexity. The complexity is very high, especially for an array with a large number of elements [7].

### 3.2. TR-DFE

**Figure 2:**
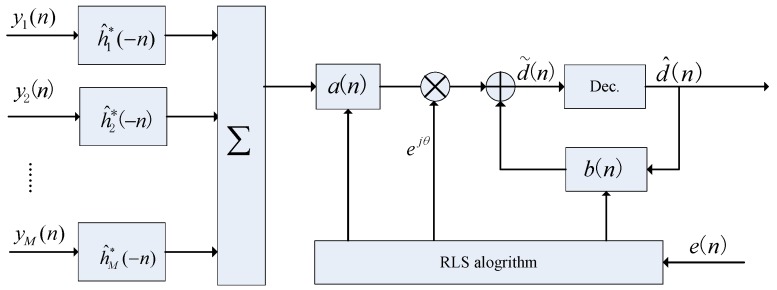
Conventional time reversal DFE (TR-DFE) block diagram.

As shown in Figure 2, the estimated channels are time reversed and conjugated, convolved with the distorted signals, and summed across branches to generate a single-channel signal with slight ISI. The spatial/temporal compressions of the TR processing reduce ISI and increase SNR. TR alone usually cannot achieve perfect equalization with a limited number of hydrophones. To compensate for residual ISI, the original TR structure is followed by a single-channel equalizer (TR-DFE). Figure 2 depicts a conventional TR-DFE system with one transmitter and one *M* receiver. TR-DFE may be viewed as a low-complexity MC-DFE as in [2]. Like MC-DFE, TR-DFE also has performed successfully in different UWA channels. In Figure 2a, r(t) denotes the combination signal after TR processing:

(6) $$r\left(t\right)=\sum_{m=1}^{M}y_{m}\left(t\right)⊗{\hat{h}}_{m}^{*}\left(-t\right),$$

where ⊗ stands for linear convolution and

(7) $$y_{m}\left(t\right)=s\left(t\right)⊗h_{m}\left(t\right)+w_{m}\left(t\right).$$

Then, the receive signal can be rewritten as follows:

(8) $$r\left(t\right)=\hat{q}\left(t\right)⊗s\left(t\right)+\tilde{w}\left(t\right),$$

where

(9) $$\hat{q}\left(t\right)=\sum_{m=1}^{M}h_{m}\left(t\right)⊗{\hat{h}}_{m}^{*}\left(-t\right),$$

and

(10) $$\tilde{w}\left(t\right)=\sum_{m=1}^{M}{\hat{h}}_{m}^{*}\left(-t\right)⊗w\left(t\right).$$

$\hat{q}\left(t\right)$ is the estimated autocorrelation of the impulse response functions summed over the channels, and $\tilde{w}\left(t\right)$ is the filtered noise. When M is large enough, $\hat{q}\left(t\right)$ is close to δ(t). The output of TR processing r(t) is equalized by the following single-channel DFE to remove residual ISI.

### 3.3. TR-MC-DFE

**Figure 3:**
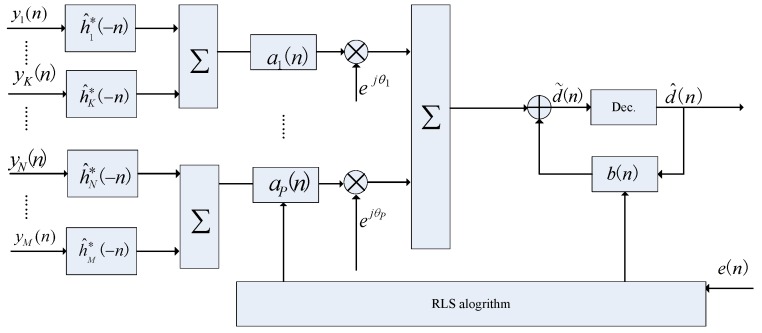
Proposed TR-DFE block diagram.

Subarray processing can improve the performance of element-domain MC-DFE [6]. In some underwater platforms, small arrays with 6–8 elements may be possible for underwater acoustic communication [11]. In this section, TR-MC-DFE is proposed to improve the performance of the TR-DFE. In this system, the entire receive array is divided into several subarrays. The proposed receiver structure is shown in Figure 3. In Figure 3a, $r_{k}\left(t\right)$ denotes the combination signal after TR processing as follows:

(11) $$r_{k}\left(t\right)=\sum_{m=1}^{K}y_{m}\left(t\right)⊗{\hat{h}}_{m}^{*}\left(-t\right),\;\;k=1,\cdots Q,$$

where *K* is the number of sensors per subarray, *Q* is the number of subarrays, and M=KQ.

Prior to the following MC-DFE, TR processing is conducted in each subarray to shorten the underwater acoustic channels. Because Q≪M, the parameters of the following low-order MC-DFE are reduced significantly.

### 3.4. Channel Estimation

The *m*-th channel received signal $y_{m}\left(n\right)$, which is Doppler-corrected, can be expressed in matrix form as follows:

(12) $$y_{m}\left(n\right)=S\left(n\right)h_{m}\left(n\right)+w\left(n\right),$$

where

(13) $$y_{m}\left(n\right)={\left[y_{m}\left(n\right)\;\cdots y_{m}\left(n-1\right)\;\cdots y_{m}\left(n-L+1\right)\right]}^{T},$$

(14) $$h_{m}\left(n\right)={\left[h_{m}\left(n,0\right)\;\cdots h_{m}\left(n,1\right)\;\cdots h_{m}\left(n,L-1\right)\right]}^{T},$$

(15) $$S\left(n\right)=\left[\begin{array}{cccc} s(n) & s(n-1) & \cdots & s(n-L+1)\\ s(n-1) & s(n-2) & \cdots & s(n-L+2)\\ \vdots & \vdots & \vdots & \vdots \\ s(n-L+1) & s(n-L+2) & \cdots & s(n-2L+2) \end{array}\right].$$

Channel estimation plays a critical role in TR underwater acoustic communications. The least square (LS), orthogonal matching pursuit (OMP), and improved proportionate normalized least mean squares (IPNLMS) are implemented to solve the above equations, and their performances are compared in this paper. UWA channels are sometimes sparse. The LS algorithm cannot take advantage of the sparsity inherent in UWA channels, which leads to a high mean squared error. The OMP algorithm is a sparse channel estimation; however, the sparsity of the channel is a necessary factor in this algorithm. The LS and OMP algorithms can be found in [16,17]. Sparseness was taken into account to apply the IPNLMS algorithm to UWA channel estimation [21,23]. The IPNLMS algorithm shows not only robust performance in non-sparse channels but also better performance than LMS in sparse channels. To utilize the sparse nature of the UWA channel, the IPNLMS adapts the coefficients $h_{m}$ as follows:

(16) $${\hat{h}}_{m}\left(n+1\right)={\hat{h}}_{m}\left(n\right)+\frac{\mu e_{m}{\left(n\right)}^{*}\Phi_{m}\left(n\right)y_{m}\left(n\right)}{y_{m}^{H}\left(n\right)\Phi_{m}\left(n\right)y_{m}\left(n\right)+\delta },$$

where (·) represents the conjugate, μ is the step size, and δ and ε are regulation parameters. $\Phi_{m}\left(n\right)$ is a diagonal proportionate matrix:

(17) $$\Phi_{m}\left(n\right)=\mathrm{diag}\left\{\phi_{m,0}\left(n\right),\cdots ,\phi_{m,L-1}\left(n\right)\right\}.$$

To guarantee its robustness in various UWA channels, the diagonal elements of $\Phi_{m}\left(n\right)$ are delineated as follows:

(18) $$\phi_{m,k}=\frac{1-\alpha }{2L}+\left(1+\alpha \right)\frac{|{\hat{h}}_{m}\left(n,k\right)|}{2∥{\hat{h}}_{m}{\left(n\right)∥}_{1}+\varepsilon },\;k=0,\cdots ,L-1.$$

## 4. Simulation Results

We have tested the statistical performance of the proposed method over 500 quasi-static fading channels. We consider a quasi-static fading model in which the channel is fixed during each packet and is independently random from packet to packet. Each packet includes 10,366 quadrature phase shift keying (QPSK) symbols, and eight received hydrophones are used. A sparse channel with an extended delay (L=100) is used in the simulation, where $N_{p}=15$ is active and the other multipath magnitudes are zero. The amplitude of the path is a Rayleigh distribution, and the average power decreases exponentially along with delay, where the first and last paths differ by 20 dB. The number of feedforward and feedback filters are 32 and 32, respectively. The fractionally spaced equalizer (two samples per symbol) is used for the feedforward filter. The RLS forgetting factor λ in the MC-DFE is 0.999. The residual phase offset is corrected by a DPLL embedded in the MC-DFE. Both $K_{1}$ and $K_{2}$ in DPLL are 0.005.

**Figure 4:**
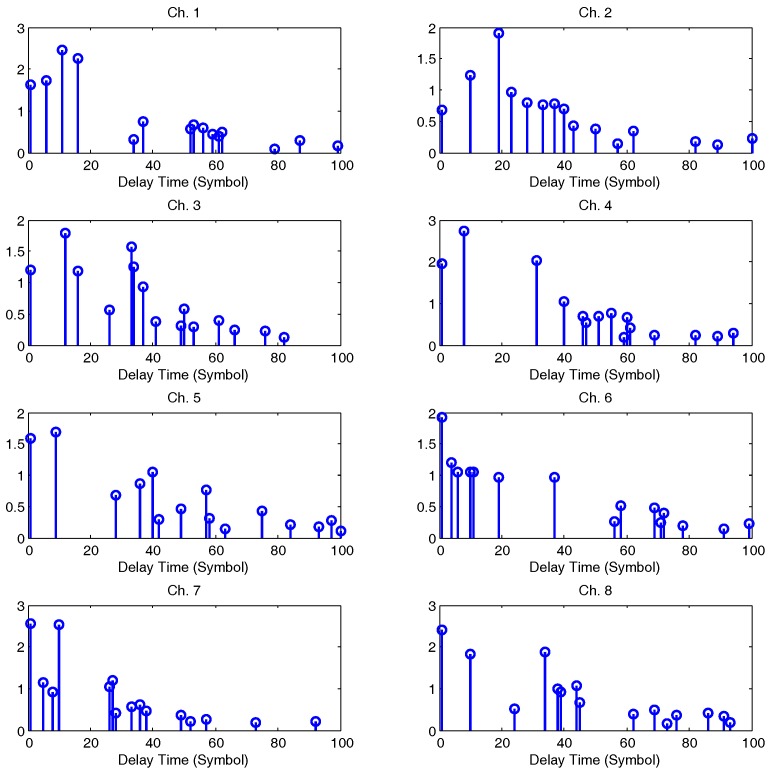
Channel impulse responses (CIR) snapshots in simulation.

**Figure 5:**
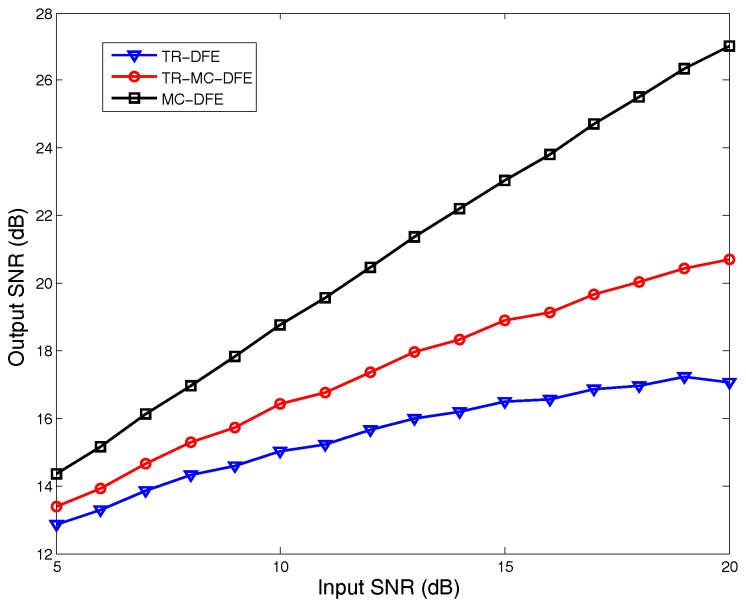
Output signal-to-noise (SNR) varies with input SNR.

Figure 4 plots the channel impulse responses (CIR) for eight channels in one simulation. Figure 5 illustrates the output SNR improvements using the proposed TR-MC-DFE according to different input SNRs. The output SNR increases as the input SNR increases. For example, when an input SNR equals 10 dB, the output SNR improvement is about 1 dB. When the input SNR equals 15 dB, the output SNR improvement is about 1.8 dB. The output SNR of the MC-DFE is higher than the TR-MC-DFE in our simulation, but its complexity for eight receivers is high.

## 5. Lake Experiment

We start by describing the 2015 Danjiangkou (DJK15) experiment in which QPSK signals are transmitted from one source to eight receivers. BER and output SNR performances are obtained by applying different algorithms to the DJK15 data. The relationship between output SNR and the number of subarrays is studied using the experimental data.

### 5.1. Experiment Setup

**Figure 6:**
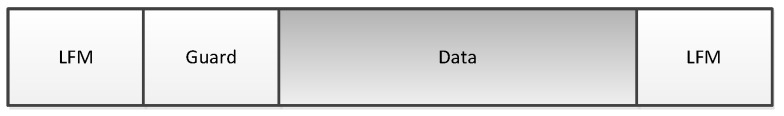
Transmitted signal structure.

The DJK15 experiment was conducted in Danjiangkou River, Henan Province, China, in January 2015. The water depth was 45–50 m. The source is deployed at 20 m below the surface. The receiver array was deployed at depths between 18–20 m. It consists of two subarrays, each with four hydrophones uniformly spaced at 0.25 m. Two subarrays are soft connected. The top hydrophones (1,2,3,4) belong to the first subarray, and the bottom hydrophones (5,6,7,8) belong to the second subarray. The data were transmitted from the source using QPSK modulation with a carrier frequency of 6 kHz and a bandwidth of 4 kHz. The structured signal was transmitted as shown in Figure 6. The transmitted signal structure was a 0.1 s linear frequency modulated signal and 0.1 s guard period followed by the data.

**Figure 7:**
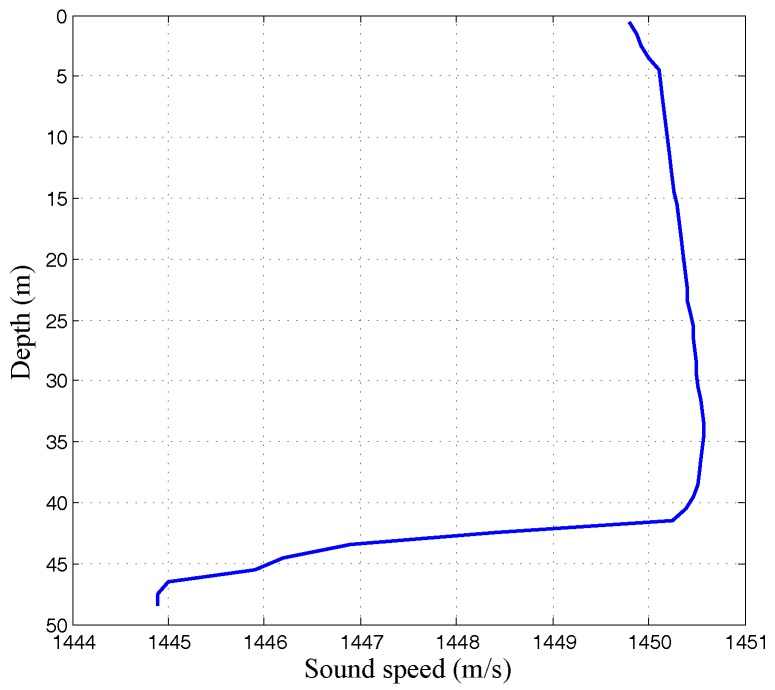
Sound speed profile in DJK15 lake experiment.

The distance between transmitter and receiver was 7.4 km. The data rate is 8 kbits/s with QPSK modulation for a symbol rate of 4 k-symbols/s. Each packet was 2.8 s, including 10,366 QPSK symbols, of which 500 symbols were training sequences. Ten packets were transmitted during that experiment. In total, the transmitted signal contained 10,366 × 10 × 2 = 20,7320 information bits. The sound speed profile was plotted at a function of depth, as shown in Figure 7.

The detail parameters of the communication system used in the lake experiment are shown in Table 1. The recursive least squares (RLS) algorithm is used for the adaptive MC-DFE. The order of feedforward and feedback filter were 50 and 50, respectively. The other parameters in the DFE and DPLL are the same as the simulation.

**Figure 8:**
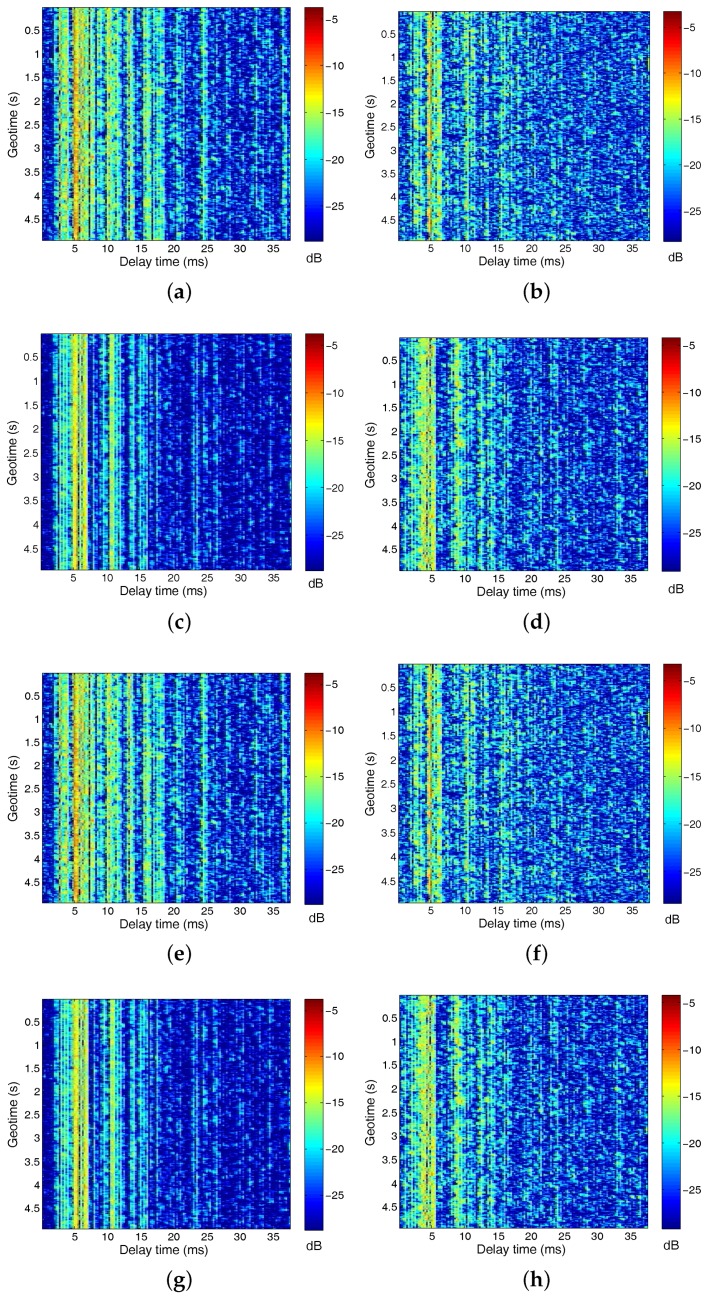
Examples of CIRs estimated using the least mean squares (LMS) algorithm from the DJK15 data: The 20 ms delay spread in the channel amounted to ISI spanning about L = 80 symbols.

**Figure 9:**
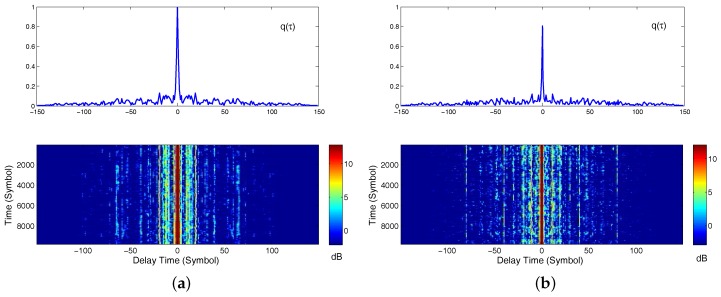
The normalized *q* function based on estimated CIRs using the LMS channel estimation algorithm: (**a**) *q* function using the channels (1,2,3,4); (**b**) *q* function using the channels (5,6,7,8). The top panel in each figure shows a snapshot of the *q* function.

The received SNRs of hydrophones (1,2,3,4) and hydrophones (5,6,7,8) was about 7 dB and 2 dB, respectively. The second subarray was rotated by the water current, and hence, it may not point to the transmitter. This is the reason that the received SNR of the second subarray is lower than the first one. The DJK15 channels varied slowly with time. Examples of CIRs for the transmissions from the source to the top and bottom receivers are shown in Figure 8. There were approximately 20 ms of delay spread in the channel, which amounted to ISI spanning about L = 80 symbols. Examples of CIRs are shown as a function of geotime in Figure 8a–h for the transmissions from the source to receivers 1–8. The top receiver is sensor one. For this receiver, the multipath structures remain relatively stable over a 5 s duration. Figure 9 plots the *q* functions for different subarrays using the measured CIRs from the DJK15 experiment. The top panel in each figure shows a snapshot of the normalized *q* function.

### 5.2. Results

The performance metric is the uncoded BER and the output SNR. The proposed TR-DFE uses two subarrays with sensors (1,2,3,4) and (5,6,7,8). Table 2 illustrates the BER results of 10 packets using different receiver algorithms. The conventional TR-DFE achieved BERs between $1\times 10^{-2}$ and $1\times \;10^{-1}$, while the proposed TR-MC-DFE achieved BERs between $3\times 10^{-4}$ and $1\times 10^{-2}$. Compared with the conventional TR-DFE, the average BER of TR-MC-DFE was reduced by an order of magnitude. The OMP and IPNLMS sparse channel estimation algorithms further improved the BER performance as shown in Table 2.

**Figure 10:**
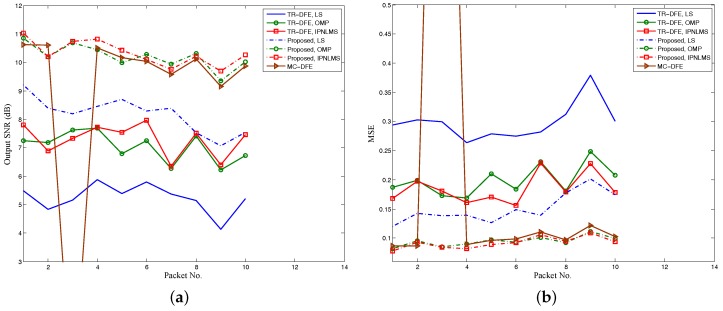
Performance for 10 packets using different methods. Results of the conventional TR-DFE are represented by solid lines. Results of the proposed method are represented by dot-dashed lines: (**a**) output SNR performance and (**b**) Mean squared error (MSE) performance.

The output SNR and MSE of 10 packets for the DJK15 experiment with different receiver algorithms are shown in Figure 10. The conventional TR-DFE achieved output SNRs between 4 dB and 8 dB, while the proposed TR-MC-DFE achieved output SNRs between 7 dB and 11 dB. The output SNR improvements obtained from subarray processing were about 3 dB, with the same for three channel estimation algorithms. For comparison, the output SNR performance of the MC-DFE was also plotted in Figure 10. The output SNR of MC-DFE was very close to that of the TR-MC-DFE except for packet 3, which failed to decode successfully. However, the computational complexity of the 8-channel DFE is much higher than TR-DFE and TR-MC-DFE, when the above parameters are selected.

**Figure 11:**
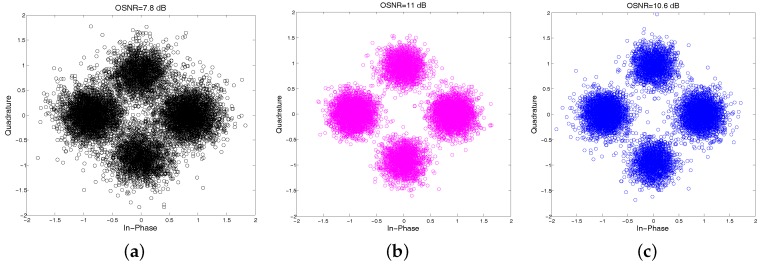
Scatter plot of the equalized first packet signal: (**a**) the conventional TR-DFE, (**b**) the proposed TR-MC-DFE (*P* = 2) and (**c**) MC-DFE.

To illustrate the improvement of the proposed TR-MC-DFE over the conventional TR-DFE, we chose the first packet among 10 packets to present the scatter plot of equalized QPSK signals using the IPNLMS channel estimation algorithm shown in Figure 11, which includes the following: (a) conventional TR-DFE; (b) TR-MC-DFE; and (c) MC-DFE. The output SNRs are 7.8 dB, 11 dB, and 10.6 dB, with the corresponding bit error rates of $1.35\times 10^{-2}$, $3.0\times 10^{-4}$, and $1.0\times 10^{-3}$, respectively. Using the conventional TR-DFE with IPNLMS, the average uncoded BER and output SNR for 10 packets are $2.1\times 10^{-2}$ and 7.3 dB, while using the proposed TR-MC-DFE with IPNLMS, the average uncoded BER and output SNR for 10 packets are $1.5\times 10^{-3}$ and 10.3 dB . The average output SNR improvement is 3 dB , whereas the maximum output SNR improvement is 3.5 dB among 10 packets.

**Figure 12:**
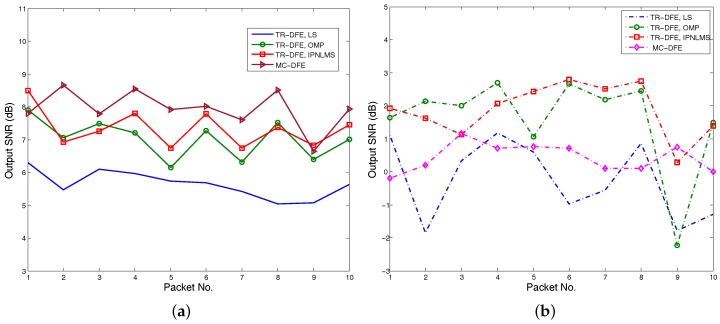
Performance of conventional TR-DFE with different subarray: (**a**) subarray with hydrophones (1,2,3,4) and (**b**) subarray with hydrophones (5,6,7,8).

Figure 12a,b show the output SNRs using hydrophones (1,2,3,4) and hydrophones (5,6,7,8) with LS, OMP, IPLNLMS and MC-DFE, respectively. The TR-DFE receiver using second hydrophones fails to demodulate because of the low SNRs shown in Figure 12b. Comparing Figure 10a and Figure 12a, the hydrophones (5,6,7,8) have no contribution to the output SNR improvement of the conventional TR-DFE. However, the hydrophones (5,6,7,8) still have contribution to output SNR improvement of the proposed TR-MC-DFE shown in Figure 10.

**Figure 13:**
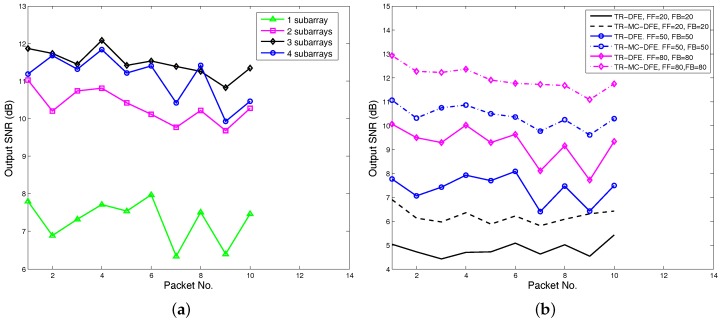
Trade-off between subarray length and DFE order. (**a**) performance of TR-MC-DFE using different subarrays, (**b**) performance of TR-MC-DFE using different DFE order.

For fixed-length arrays, TR-MC-DFE with different subarray lengths (number of subarrays) have different performance and computational complexity. A trade-off needs to be made between computational complexity and receiver performance. Meanwhile, a minimum subarray length is required to obtain a acceptable Q function (ISI). The output SNR performance using TR-MC-DFE with four different subarrays is shown in Figure 13a. The feedforward and backward filter order are 50 and 50, and IPNLMS is used. For one subarray, the TR-MC-DFE becomes the conventional TR-DFE and eight hydrophones are used. For two subarrays, we use hydrophones (1,2,3,4) and hydrophones (5,6,7,8). For three subarrays, we use hydrophones pairs (1,2,3), (5,6,7), and (6,7,8). For four subarrays, (1,2), (3,4), (5,6), and (7,8) hydrophones are used. As shown in Figure 13a, from one subarray to two subarrays, the average output SNR gain is about 3 dB, while the average output SNR gain drops to 1.2 dB from two subarrays to three subarrays. In particular, from three subarrays to four subarrays, the output signal to noise ratio even decreased by 0.4 dB. From DJK15 data, it is recommended that each subarray use 3-4 hydrophones. For a fixed-length array, reducing the subarray length leads to more subarrays. More subarrays increase the channel number of the following MC-DFE, thereby increasing the computational complexity.

Figure 13b shows the output SNR improvements with different DFE orders. IPNLMS channel estimation algorithms are used, and two subarrays are used in the TR-MC-DFE. Both TR-DFE and TR-MC-DFE have output SNR improvements when the orders of DFE increase. In practice, the choice of DFE order depends on the Q function and the processor’s capabilities. As shown in Figure 9, we can choose a value 10 dB smaller than the Q function maximum for DFE order.

## 6. Conclusions

In this paper, we propose a TR-MC-DFE method that uses subarray processing for high-rate underwater acoustic communication. This method couples passive TR processing and MC-DFE. We used simulation and processed data collected in the DJK15 lake experiment to measure the performance of the proposed method. The results show that the TR-MC-DFE has the potential to improve the performance of conventional TR-DFE. Based on the lake experimental data, the output SNR can be increased by up to 3.4 dB. In these data, the output SNR gap between MC-DFE and TR-MC-DFE for lake experimental data is very small. However, the complexity of MC-DFE is much higher. Sparse channel estimation using OMP and IPNLMS algorithms can be further superior to existing systems. Trade-off between subarray length and DFE order was also investigated. The results show that the proposed method provides a possible solution for communicating with an underwater platform with 6–8 sensor elements.

## Figures and Tables

**Table 1:** System parameters.

Parameters	Description	Values
$T_{c}$	Symbol duration	0.25 ms
*M*	Total sensor numbers	8
*K*	Oversampling rate	2
$N_{p}$	The training symbol length	500
$N_{f}$	Feedforward filter order	50
$N_{b}$	Backward filter order	50
$K_{1}$	Proportional tracking constants in PLL	0.005
$K_{2}$	Intergral tracking constants in PLL	0.005
λ	RLS forgetting factor in DFE	0.999

**Table 2:** Uncoded Bit Error Rate (BER) Performance of DJK15 Experiments.

Packet Nos.	TR-DFE			TR-MC-DFE		
	LS	OMP	IPNLMS	LS	OMP	IPNLMS
1	0.054	0.0206	0.0135	0.0043	0.0007	0.0003
2	0.074	0.0194	0.0256	0.0085	0.0016	0.0025
3	0.064	0.0169	0.0192	0.011	0.0006	0.0006
4	0.044	0.0145	0.0161	0.0078	0.0011	0.0007
5	0.059	0.0246	0.0165	0.0076	0.0024	0.0012
6	0.046	0.0204	0.0127	0.0088	0.0010	0.0022
7	0.058	0.0393	0.0357	0.0085	0.0016	0.0026
8	0.063	0.0171	0.0179	0.0163	0.0013	0.0012
9	0.097	0.0354	0.0331	0.024	0.0045	0.0027
10	0.062	0.029	0.0182	0.0168	0.0016	0.0012
Average	0.062	0.0237	0.0209	0.0113	0.0016	0.0015
